# Primase-polymerases: how to make a primer from scratch

**DOI:** 10.1042/BSR20221986

**Published:** 2023-07-13

**Authors:** Lewis J. Bainbridge, Katerina Zabrady, Aidan J. Doherty

**Affiliations:** Genome Damage and Stability Centre, School of Life Sciences, University of Sussex, Brighton BN1 9RQ, U.K.

**Keywords:** CAPP, Pri1, Prim-Pol, primase, primer initiation, PrimPol

## Abstract

To pass on genetic information to the next generation, cells must faithfully replicate their genomes to provide copies for each daughter cell. To synthesise these duplicates, cells employ specialised enzymes called DNA polymerases, which rapidly and accurately replicate nucleic acid polymers. However, most polymerases lack the ability to directly initiate DNA synthesis and required specialised replicases called primases to make short polynucleotide primers, from which they then extend. Replicative primases (eukaryotes and archaea) belong to a functionally diverse enzyme superfamily known as Primase-Polymerases (Prim-Pols), with orthologues present throughout all domains of life. Characterised by a conserved catalytic Prim-Pol domain, these enzymes have evolved various roles in DNA metabolism, including DNA replication, repair, and damage tolerance. Many of these biological roles are fundamentally underpinned by the ability of Prim-Pols to generate primers *de novo*. This review examines our current understanding of the catalytic mechanisms utilised by Prim-Pols to initiate primer synthesis.

## Introduction

Before cell division can occur, a cell’s genome must be duplicated. The process of DNA replication typically begins with the synthesis of short RNA primers by specialised replicases called primases and continues with the extension of these primers by DNA polymerases. Primases can be divided into two distinct enzyme groupings, DnaG and Primase-Polymerase (Prim-Pol) superfamilies, with the latter formerly known as archaeo-eukaryotic primases (AEPs). These families display no overt sequence relationships, suggesting multiple instances of emergence throughout evolution, and can be distinguished by conserved structural features. DnaG-type primases are characterised by a topoisomerase-primase (TOPRIM) fold within their catalytic domains [1]. This fold is shared with Type IA and Type II topoisomerases, bacterial RecR/M family DNA repair proteins and archaeal OLD family nucleases. Notably, the TOPRIM fold of DnaG forms a crescent-shaped α/β fold structure that does not resemble any known DNA or RNA polymerases [2]. DnaG primases are characterised by two catalytic motifs centred around conserved acidic amino acids (E and DxD) that coordinate metal ions. These key residues reside within the core of the active site, in a concave depression on the enzyme’s surface.

**Figure 1:**
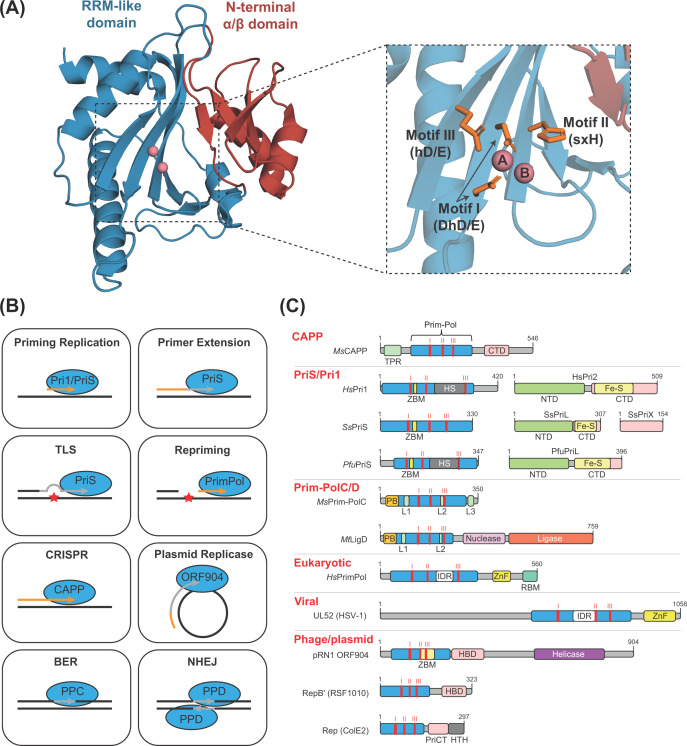
Structure and function of Prim-Pol proteins (**A**) The architecture of a Prim-Pol domain. Left: the crystal structure of the Prim-Pol domain of *Marinitoga* sp. 1137 CAPP (PDB ID: 7P9J). Right: zoomed image of the CAPP Prim-Pol domain with key residues (orange) within conserved motifs indicated. The N-terminal α/β fold, red; the RRM-like domain, blue; metal ions, pink spheres. (**B**) Examples of the diverse range of Prim-Pol (blue) functions, utilising *de novo* primer synthesis (orange) or primer extension activities (grey). TLS, translesion synthesis; CRISPR, role in CRISPR spacer acquisition; BER, base excision repair; NHEJ, non-homologous end joining; PPC, Prim-PolC; PPD, Prim-PolD. (**C**) Domain architecture of selected members of Prim-Pol superfamily. *Ms*CAPP, *Marinitoga* sp. 1137 CAPP; HsPri1/2, *Homo sapiens* Pri1/2; *Ss*PriS/L/X, *Saccharolobus solfataricus* PriS/L/X; *Pfu*PriS/L, *Pyrococcus furiosus* PriS/L; *Ms*Prim-PolC, *Mycolicibacterium smegmatis* Prim-PolC; *Mt*LigD, *Mycobacterium tuberculosis* LigD; *Hs*PrimPol, *Homo sapiens* PrimPol; UL52 (HSV-1), Herpes simplex virus type 1 UL52; pRN1 ORF904, ORF904 protein from plasmid pRN1; RepB′ (RSF1010), RepB′ protein form RSF1010 plasmid; Rep (ColE2), Rep protein from ColE2 plasmid. The core Prim-Pol domain (blue rectangle) is displayed alongside its ancillary domains. TPR, Tetratricopeptide repeat domain; CTD, C-terminal domain; ZBM, Zinc binding motif; HS, Helical subdomain; NTD, N-terminal domain; PB, Phosphate binding pocket; Fe-S, Iron-sulfur cluster; L1-3, Loop 1-3; IDR, Intrinsically disordered region; ZnF, UL52-like zinc finger domain; RBM, RPA binding motif; HBD, Helix bundle domain; HTH, Helix-turn-helix; I, II, III (red), active site motif I, II & III. Numbers represent the positions of the first and last amino acids.

In contrast, members of the Prim-Pol superfamily are structurally characterised by an N-terminal α/β fold and a C-terminal RNA recognition motif (RRM)-like fold, which together form the Prim-Pol catalytic domain (Figure 1A) [3]. The RRM-like fold is also found in viral RNA-dependent RNA polymerases and reverse transcriptases and forms the palm domain of DNA polymerases from the A, B, and Y families. Bioinformatic analyses of the Prim-Pol superfamily identified the defining characteristics of Prim-Pols and uncovered a multitude of new superfamily members divided amongst distinct clades and families [3]. Three amino acid motifs are conserved in all members of this superfamily: motif I (hhhDhD/E), II (sxH), and III (hD/E) (h, hydrophobic; s, small [AGSVCDN]; x, any). Within the RRM-like structure, the key residues of these three motifs are positioned in close proximity, permitting interactions with metal ions and nucleotides, which are requisite for catalysis [3,4].

## Diverse functions of Prim-Pol enzymes

Despite sharing conserved structural and catalytic motifs, Prim-Pols have evolved to partake in a diverse variety of roles in DNA metabolism [4]. The previous demarcation of Prim-Pols as ‘primases’ is somewhat of a misnomer since these enzymes conduct a range of functions including, but not limited to, initiating DNA replication (Figure 1B). Such functional flexibility is likely facilitated by a diverse range of domain architectures and subunit compositions that surround the core catalytic Prim-Pol domains of different members of this superfamily (Figure 1C).

Archaea rely on Prim-Pols to initiate and propagate genome replication, presumably to compensate for the absence of Pol α in these organisms. *Pyrococcus furiosus* (*Pfu*) PriS can synthesise DNA fragments of several kilobases in length [5]. *Pfu*PriS is also able to conduct translesion synthesis (TLS), accurately copying templates with oxidative or other lesions, potentially providing the archaeal replisome with an inherent mechanism for lesion bypass [6]. Furthermore, Prim-Pols have also evolved roles in extra-chromosomal plasmid replication in archaea. For instance, the archaeal cryptic plasmid pRN1 encodes the ORF904 protein, which consists of a helicase domain alongside a Prim-Pol domain that endows the enzyme with both primase and polymerase activities [7]. The combination of these domains enable ORF904 to synthesise many kilobases of DNA whilst replicating pRN1 plasmids.

Prim-Pol orthologues are also present in many bacterial species. One notable example is the CRISPR-associated Prim-Pols (CAPPs), which were discovered through their operonic association with CRISPR-Cas genes [8]. CAPPs display primase, polymerase, and synthesis-dependent strand-displacement activities, and likely undertake roles in CRISPR spacer acquisition. Prim-Pol orthologues are also found co-operonically with a prokaryotic non-homologous end-joining (NHEJ) protein called Ku [9,10], which facilitates DNA double-strand break (DSB) repair. Here, the Prim-Pol domain (PolDom/Prim-PolD) is part of a larger DNA break repair protein known as Ligase D (LigD), which forms a complex with Ku and facilitates prokaryotic NHEJ [11]. This process takes advantage of this Prim-Pol’s unusual proficiency in promoting microhomology-mediated end-joining (MMEJ), followed by subsequent gap filling if required, to facilitate DSB repair [12–14].

In mycobacteria, a Prim-PolD homologue called Prim-PolC interacts with its operonic partner Ligase C to conduct roles in excision repair pathways [15]. As part of this process, Prim-PolC binds to short gaps produced during excision processes and conducts gap-repair synthesis [16]. Notably, Prim-PolC and Prim-PolD primarily function as polymerases, not primases, since the presence of additional DNA binding motifs (Loops 1, 2, and 3 in Prim-PolC and Loops 1 and 2 in Prim-PolD) promotes preferential binding to particular dsDNA structures to encourage polymerase-mediated repair synthesis, rather than primase activity [12–14,16].

Finally, the most prominent function of Prim-Pols is the synthesis of primers to initiate DNA synthesis. For example, bacterial RepB′ and Rep proteins found on RSF1010 and ColE2 plasmids, respectively, generate short primers to initiate plasmid replication [17,18]. However, perhaps the most widely studied Prim-Pol primase is PriS (PRIM1/Pri1), which fundamentally underpins eukaryotic and archaeal DNA replication [19,20]. Two distinct Prim-Pol superfamily members can be found in most eukaryotic organisms: the replicative primase (Pri1) and Primase-Polymerase (PrimPol). Pri1 initiates DNA synthesis during canonical replication by priming Okazaki fragment formation, as part of the Pol α-primase complex, [21–23]. In contrast with Pri1, which is ubiquitously required for eukaryotic replication, PrimPol is not found in all eukaryotes and is notably absent from *Caenorhabditis elegans*, *Drosophila melanogaster*, *Saccharomyces cerevisiae*, and *Schizosaccharomyces pombe*. In accordance with its name, PrimPol has the ability to conduct both primase and polymerase synthesis activities and its major role is to reprime DNA synthesis following replication fork stalling [24–30]. PrimPol, therefore, provides a canonical mechanism of DNA damage tolerance against a variety of replication fork-stalling lesions, thereby promoting genomic stability [30]. In addition to priming, human PrimPol is also an adept TLS polymerase *in vitro*, able to bypass a variety of DNA lesions [24,28,31–33]. However, while the role of PrimPol-mediated repriming in DNA damage tolerance is well established, the physiological relevance of PrimPol’s TLS activities is currently unclear.

## Mechanism of *de novo* primer synthesis by Prim-Pols

**Figure 2:**
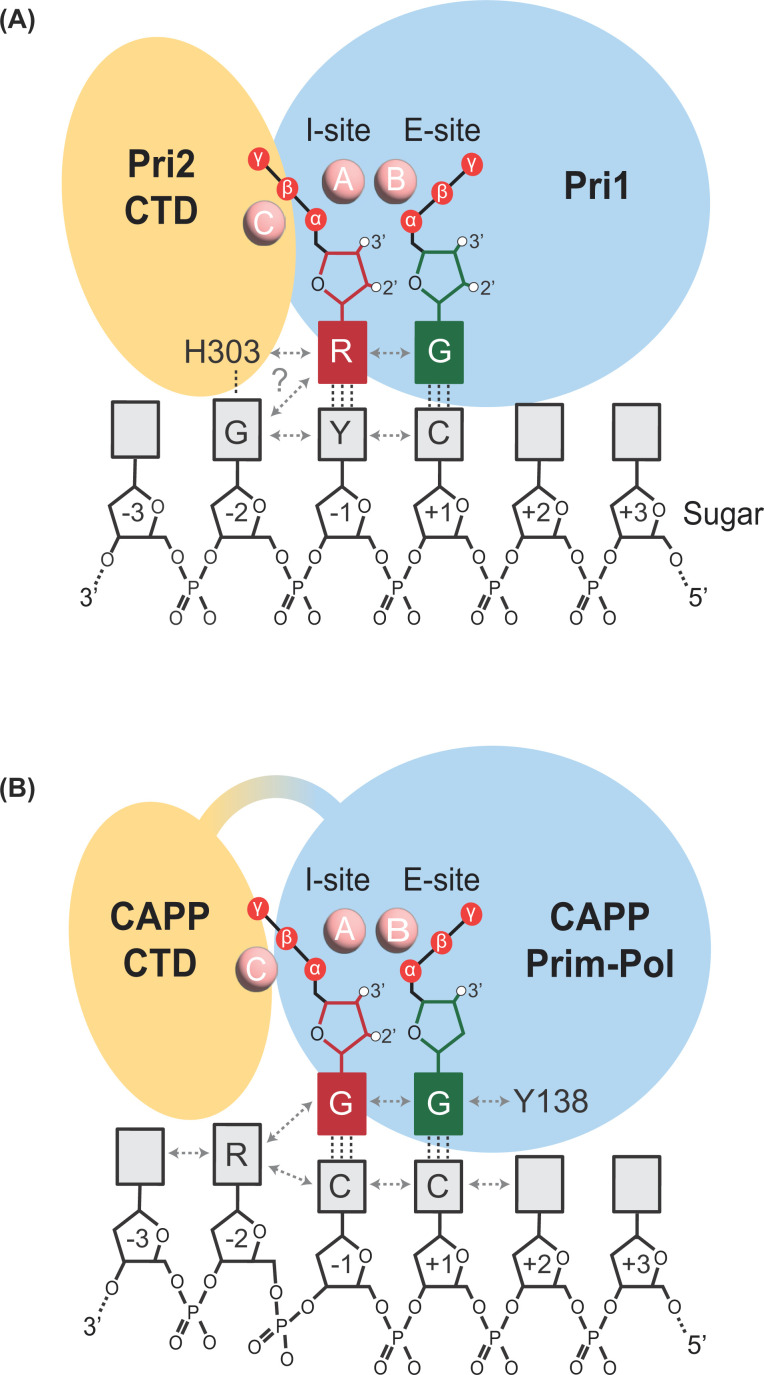
Prim-Pol primer initiation complexes (**A**) Model of human Pri1/2 primer initiation complex. Pri1 (blue) coordinates the initiating (red) and elongating (green) nucleotides in its I- and E-site, respectively, and metal ions A and B (pink spheres). The triphosphate tail of initiating nucleotide interacts with metal C (pink sphere) and is bound by Pri2 CTD (yellow). (**B**) Model of *Ms*CAPP primer initiation complex. CAPP Prim-Pol domain (blue) coordinates the initiating and elongating nucleotides and metal ions A and B. The triphosphate tail of initiating nucleotide interacts with metal C and might be bound by CAPP CTD (yellow). π–π stacking interactions (gray dashed arrow) and Watson–Crick base paring interactions (black dashed line) are displayed. Numbers from -3 to +3 represent the relative template position to the primer initiation site. Red sphere, phosphate group; white sphere, 2′/3′-OH group; grey rectangle, base; G, guanine; C, cytosine; R, purine; Y, pyrimidine; empty rectangle, any base.

To initiate *de novo* DNA synthesis, a primase must bind a single-stranded DNA (ssDNA) template and coordinate two nucleotides (initiating and elongating) and two divalent metal ions within its active site (Figure 2). Primer synthesis occurs through a biphasic process of initiation (*de novo* dinucleotide synthesis) and extension, whereby nucleotides are attached to the elongating polymer. Finally, primer synthesis is then terminated and primers are transferred to the active site of a more processive replicative polymerases for further elongation. To comprehensively examine the mechanism of primer synthesis by Prim-Pols, each step of the catalytic process is reviewed below.

## DNA binding and sequence specificity

The binding of a primase to single-stranded DNA (ssDNA) likely represents the first step of primer synthesis [34]. The ability of primases to bind to and operate specifically on ssDNA is critical for their function. Accordingly, while polymerases contain a thumb domain that contacts a dsDNA template, the catalytic domain of Prim-Pols lack an equivalent structure and, therefore, are not dependent on a pre-existing primer for DNA binding [35–37]. In the case of the mammalian Pri1/Pri2 heterodimer, ssDNA binding is shared between both subunits [38]. Similarly, human PrimPol binds ssDNA via its Prim-Pol domain and probably also using its zinc finger domain [26]. DNA binding by eukaryotic PrimPol’s catalytic core is primarily mediated by two DNA binding motifs (Motif Ia and Motif Ib) located in the N-terminal region of the enzyme [37]. The Prim-Pol domain of bacterial CAPPs binds DNA using motifs analogous to human PrimPol [39]. The DNA binding step of primer synthesis is thought to be metal-independent [34,40].

Primases display a preference for the templating sequence upon which they initiate primer synthesis *in vitro* [19,20]. Early studies investigating mammalian replicative primases noted that the enzyme displayed a preference for binding to polypyrimidine template sequences, suggesting that the selection of the initiation site for primer synthesis is not random [41]. Furthermore, ATP and/or GTP are required for the efficient initiation of primer synthesis, while CTP and UTP only minimally affect activity, suggesting that pyrimidine templates are required to support efficient initiation with purine nucleotides [41–44]. The nucleotide that will become the second base of a primer was found to have the largest effect on initiation site selection and, due to this, altering the concentrations of nucleotides can alter the selected initiation site [34]. For Pri1, a strong preference was observed for GTP in this position, which is likely due to the extra stability provided by the additional hydrogen bond in G-C base pairing compared with A-T. An equal preference for either purine nucleotide as the initiating (5′) base was observed. One study investigating the mouse primase noted that preferential sites for primer initiation contained a guanine base next to the 5′ side of the initiating template base (-2 position), which is not copied [45]. Together, these studies imply that Pri1 preferentially initiates primer synthesis upon 3′-G-Y-C-5′ template sequences, where the pyrimidine base (Y) permits the use of a complementary purine (R) nucleotide as the initiating nucleotide of the primer. It has been proposed that, following DNA binding and prior to primer initiation, the replicative primase conducts low-affinity template sliding to locate a preferential template sequence [34]. Consistent with the findings of *in vitro* analyses, *in vivo* studies on priming have also observed a preference for initiating primer synthesis with purine nucleotides, at sites containing a guanine base in the -2 position [45,46].

Herpes simplex virus type 1 (HSV-1) UL52 is a primase subunit of the helicase-primase complex UL5-UL8-UL52 [47]. UL52 primase displays initiation site preferences similar to the related mammalian replicative primase. For instance, primers are typically initiated with a purine nucleotide, with ATP utilised more frequently [48]. Accordingly, increasing the concentration of GTP or ATP increased the rate of primer synthesis, whereas altering the concentration of CTP or UTP had almost no effect [49]. A preference for dGMP at the -2 template position was also reported, which enhances the extension of primers [48,50]. The preferential template sequence for primer initiation by HSV-1 UL52 primase was therefore concluded to be 3′-G-Y-Y-5′ [48].

Both *Dysgonamonadaceae bacterium* (*Db*) and *Marinitoga piezophila* (*Mp*) CAPP display a strong preference for initiating primers with GTP [8]. Weaker stimulation of priming was observed with ATP, while CTP and UTP had minimal effect, suggesting that CAPPs also preferentially initiate primer synthesis with a 5′ purine nucleotide. Furthermore, priming by these enzymes was most efficient when dGTP could be utilised as the second base of the primer, with optimal activity observed on 3′-C-C-5′ template sequences. Additionally, a preference for a purine base at the -2 template position was observed for *Marinitoga* sp. 1137 (*Ms*) CAPP (>98% identical with *Mp*CAPP) [39]. Together, these findings identify 3′-R-C-C-5′ as the preferential template sequence for primer initiation by CAPP.

While the template specificity of human PrimPol has not been comprehensively investigated, studies have demonstrated that the enzyme can prime efficiently on the same sequence as HSV-1 UL52 (3′-G-T-C-5′) [40]. The -2 position guanine base in this sequence was required for the most efficient primer synthesis, and this was dependent on the zinc finger domain. However, this study did not compare the priming efficiency of PrimPol with alternative initiating nucleotides. A more recent study reported that PrimPol prefers GTP over ATP as the primer-initiating nucleotide, and dGMP or dAMP in the -2 position of the template stimulated primer initiation *in vitro* [39]. In conclusion, the preferred template sequence for primer initiating by human PrimPol is most likely the same as CAPP’s - 3′-R-C-C-5′.

## Two nucleotide binding sites

Once the primase is bound to a ssDNA template, two nucleotides (initiating and elongating), and divalent metal ions must be coordinated within the active site to facilitate dinucleotide bond formation. A key feature of primases is the presence of two nucleotide-binding sites; an initiation site (I-site) and an elongation site (E-site) [51]. The I-site binds the initiating nucleotide that will form the 5′ terminus of the primer during dinucleotide synthesis, while the E-site binds the elongating nucleotide to be added to the 3′ end. At the core of these binding sites resides two divalent metal ions coordinated by the conserved catalytic triad of acidic residues from Prim-Pol’s motifs I and III [3] (Figure 2). During the coordination of nucleotides, metal A interacts with both nucleotides, while metal B exclusively interacts with the E-site nucleotide [39].

The available structural models of Prim-Pol primases have revealed that the E-site is located in the core Prim-Pol domain and consists of a depression of basic/polar residues [36,37,39]. The conserved residues of Motif II, which are characteristic of Prim-Pol superfamily enzymes, form a key part of this nucleotide binding site [3]. Conversely, the location of the I-site has proven to be more enigmatic. All primase-proficient Prim-Pols examined to date have ancillary domains or subunits associated with the core Prim-Pol domain. In the case of eukaryotic Pri1/Pri2, nucleotides were found to predominantly cross-link to Pri1 [52]. Pri1 exhibits primase activity without Pri2, however, this activity is strongly stimulated in the presence of Pri2 [39,53,54]. Structural studies have suggested that the initiating nucleotide in the I-site of Pri1 is likely stabilised by the Pri2 subunit [55,56] (Figure 2A). In the archaeal primase from *Sulfolobus solfataricus* (*Ss*) *Ss*PriX, a subunit (PriX) structurally homologous to the eukaryotic Pri2 C-terminal domain (CTD), appears to play an analogous role to human Pri2 in the stabilisation of the initiating nucleotide within the I-site of PriS [56–58].

Recently, the structure of a Prim-Pol ternary complex was determined for *Ms*CAPP's Prim-Pol domain revealing, for the first time, the molecular contacts between a DNA primase and two nucleotides (initiating and elongating) during primer initiation [39]. Crucially, all the molecular determinants of primer synthesis were found to reside within the catalytic core, consistent with functional studies of *Mp*CAPP’s activities. Although, modelling of a full-length CAPP Prim-Pol primer initiation complex identified putative interactions between the CTD and initiating nucleotide suggesting that the CAPP CTD plays an analogous role to Pri2/PriX (Figure 2B). However, CAPP’s CTD has fewer potential contacts with the initiating nucleotide than Pri2/PriX, explaining why the CAPP Prim-Pol domain is an efficient primase without the CTD domain *in vitro* [56]. Although the structure of a human PrimPol primer-initiation complex is not yet available, the structural conservation between the Prim-Pol domains of CAPP and human PrimPol enabled the identification of the I-site in the PrimPol catalytic domain [39]. Similar to CAPP, the catalytic core of PrimPol, without its ancillary zinc finger domain, can also prime efficiently [39]. However, PrimPol’s priming activity is stimulated in the presence of its UL52-like zinc finger domain, leading to a proposal that the zinc finger domain may play a similar role to Pri2, although these domains are structurally distinct [40,59].

## E-site nucleotide binding

Following the binding of a Prim-Pol primase to DNA, the initiating and elongating nucleotides are thought to bind within the active site in an ordered manner [34,39]. The first nucleotide to bind is the E-site nucleotide (elongating nucleotide), which will become the second nucleotide incorporated during *de novo* primer synthesis. Notably, Prim-Pol’s have been observed to bind dNTPs in their E-sites in the absence of DNA, suggesting that nucleotide binding could potentially precede DNA binding [13,39,60].

Sugar selectivity for the E-site nucleotide varies between Prim-Pols. For instance, human replicative primase Pri1 preferentially utilises NTPs for priming, which is facilitated by interactions between the 2′-hydroxyl group and E-site residues [36]. It has been proposed that archaeal primase *Ss*PriS also preferentially coordinates NTPs in the E-site in cells [61]. However, this is not due to an increase in binding affinity for NTPs, compared with dNTPs. In fact, *Ss*PriS binds dNTPs with a higher affinity than NTPs. Instead, the higher physiological concentrations of NTPs in these organisms, compared with dNTPs, likely favours the usage of NTPs [62]. Hydrogen bonding may also favourably stabilise or position the 2′-hydroxyl group, similar to human Pri1 [57]. In contrast with PriS/Pri1, both CAPP and human PrimPol strongly select for dNTPs as the second base in nascent primers due to a steric gate residue within their E-sites [8,24,25,63]. In human PrimPol, sugar selectivity is ensured by a conserved tyrosine (Y100), which poses a steric hindrance to the 2′-hydroxyl group of NTPs [63]. Mutating this steric gate residue to histidine (PrimPol^Y100H^) ablates E-site sugar selectivity to generate a DNA-dependent DNA/RNA primase.

## I-site nucleotide binding

The binding of the I-site nucleotide occurs following the formation of the E:DNA:(d)NTP complex, which represents the final substrate binding step during primer initiation [34,39]. The affinity of the mammalian replicative primase for the I-site nucleotide is thought to be an order of magnitude lower than that of the E-site nucleotide [52]. Similar findings have been observed for the Prim-Pol domain of CAPP [39]. Furthermore, similar to the E-site nucleotide (dATP or dGTP), the I-site nucleotide (GTP) alone does not stimulate DNA binding by CAPP. Notably, however, DNA binding affinity increased significantly upon the addition of both E-site and I-site nucleotides in the presence of divalent metal ions. This suggests a cooperative model of substrate binding that stabilises the primer initiation complex to facilitate primer synthesis. An interaction between human PrimPol and an initiating nucleotide has not yet been observed, suggesting that the interaction between the I-site and the initiating nucleotide is weak and transient [40].

The weak binding of Prim-Pols to the I-site nucleotide is likely a result of minimal contacts between the Prim-Pol domain and the initiating nucleotide. Therefore, to enable efficient primer synthesis, additional stabilising interactions are formed. It has been suggested that during primer initiation by human primase Pri1, Pri2 CTD interacts with the triphosphate group of the initiating NTP (Figure 2A) [36,64]. Additionally, the I-site base is likely supported by stacking interactions with a histidine residue on Pri2 [55,56]. A third metal ion (metal C) binds to the triphosphate tail of the initiating nucleotide. This may keep the nucleotide triphosphate tail in a conformation which enables interaction with Pri2 CTD and also acts to neutralise repulsive forces between triphosphate tails of initiating and elongating nucleotides.

The crystal structure of a CAPP primer initiation complex was determined without ancillary domains [39]. This revealed that the initiating nucleotide is held in place by metal ions, interactions with the Prim-Pol domain, Watson–Crick base paring with template bases, and a network of π–π stacking interactions between template, initiating and elongating nucleotides, and a tyrosine residue (Y138), which interacts with the base of the elongating nucleotide (Figure 2B). The efficiency of primer synthesis is greatest when a purine base is at the -2 position of the template, suggesting that pyrimidine bases are less efficient at stabilising the I-site nucleotide due to their smaller size and therefore lower potential to create π–π interactions with the initiating nucleotide [39], as described for RNA-dependent RNA polymerases [65].

Human PrimPol has been reported to prime most efficiently when guanine is present at the -2 position of the template [40]. This stimulation was dependent on the presence of the zinc finger domain, which is thought to bind the triphosphate tail of the initiating nucleotide in a manner similar to Pri2. However, another study showed that a PrimPol truncation, lacking the zinc finger domain, is stimulated by purine nucleotides in the template -2 position, similar to CAPP, suggesting that an analogous π–π network is formed within the catalytic core of the PrimPol primer preinitiation complex [39]. Without additional structural information, the exact mechanism of stimulation by a guanine/purine base at the -2 template position, and the role of the zinc finger domain in this, remain unclear.

Sugar selectivity is also displayed during I-site nucleotide binding. CAPP exclusively initiates primers with a ribonucleotide and, despite displaying strict dNTP selection in the E-site, is inefficient at initiating synthesis with dNTPs alone [8]. Here, the 2′-hydroxyl group is coordinated by the A-site metal ion and a glutamic acid residue (E260) [39]. Similar biochemical properties have also been observed for the archaeal plasmid replicase ORF904 [66]. Both *Ss*PriSLX and eukaryotic Pri1/2 complexes efficiently conduct primer synthesis using NTPs, implying that NTPs are readily utilised as the I-site nucleotide [19,34,52,57]. Structural modelling has implied that interactions between the 2′-hydroxyl group of the initiating nucleotide with metal A and aspartate 306 (D306) also underpins sugar selectivity for ribonucleotides in the I-site of Pri1; however, further structural studies are required to confirm this [67]. Human PrimPol can efficiently utilise either an NTP or dNTP as the I-site nucleotide, with a slight preference for NTPs observed *in vitro* [40]. Given that the physiological concentration of ribonucleotides are much higher than dNTPs [68], it is likely that PrimPol initiates primer synthesis using a single NTP in cells.

## Dinucleotide synthesis

**Figure 3:**
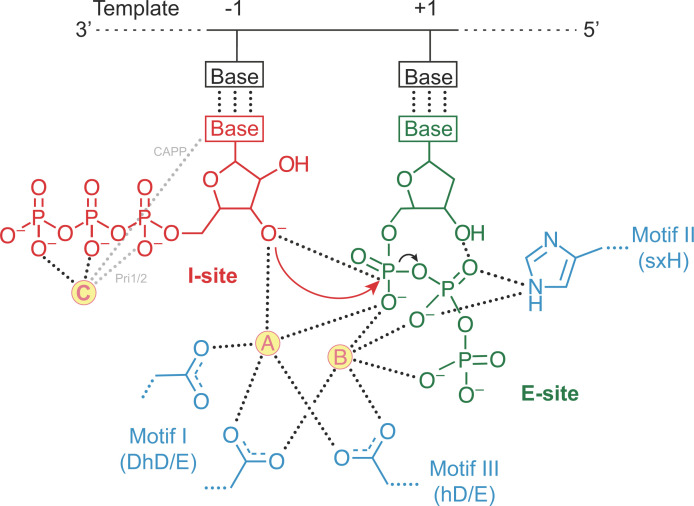
Mechanism of Prim-Pol-dependent catalysis of primer initiation Metal A and B (yellow spheres) are coordinated by a catalytic triad of residues from motif I and III (blue) in the active site of the Prim-Pol domain. Metal A interacts with the I-site nucleotide (red) and the E-site nucleotide (green). Metal B interacts with the phosphate tail of the E-site nucleotide. Metal A facilitates the nucleophilic attack of the 3′-OH group of initiating nucleotide on the α-phosphate of the elongating nucleotide. Metal B chelates one oxygen from each phosphate of the elongating nucleotide, stabilises the negative charge of the leaving oxygen, and also facilitates the release of pyrophosphate. Metal C (yellow sphere) coordinates the triphosphate tail of the initiating nucleotide. Hydrogen bonds are presented as dashed lines. Grey dash lines represent hydrogen interactions that differ between proteins. The red arrow indicates bonds formed and the black arrow indicates bonds broken during catalysis.

Once the E:DNA:NTP:(d)NTP complex is established, dinucleotide synthesis can proceed. Following the formation of a primer initiation complex, Prim-Pol primases experience the highest affinity for DNA, suggesting that they tightly lock onto the template with the initiating nucleotides correctly positioned [39]. Primer synthesis then proceeds with the formation of a phosphodiester bond between the I-site and E-site nucleotides to produce a dinucleotide. Crucially, this reaction is dependent on the presence of two divalent metal ions in the active site. Mutation of Motif I or III residues comprising the catalytic triad ablates the catalytic activity of Prim-Pols by preventing metal binding [8,24,29,39,69]. Interestingly, although the structural fold of the core Prim-Pol domain is distinct from that of polymerases, the positioning of the three invariant catalytic residues is superposable onto those of the X-family polymerase Pol β, suggesting that the proposed two-metal ion model of polymerisation also applies to Prim-Pols (Figure 3) [70,71]. In this model, the two metal ions form important interactions with the two nucleotide substrates. Metal A interacts with the 3′-hydroxyl moiety of the primer strand to lower the pK_a_ of the hydroxyl group in preparation for nucleophilic attack, while metal B interacts with the phosphate tail of the incoming dNTP and stabilises the negative charge on the leaving oxygen. Metal B is also predicted to facilitate the leaving of the phosphate groups so that the polymerase can translocate to synthesise the next nucleotide. The rate-limiting step of the reaction is thought to occur before or during the process of dinucleotide bond formation [34,49]. Following enzymatic turnover, the affinity of the enzyme for DNA decreases, probably due to the cleavage of pyrophosphate from the E-site nucleotide, which significantly decreases contacts of the E-site nucleotide with the enzyme, permitting translocation of the primase along the DNA template [39]. Notably, it has been suggested that a third metal ion (metal C) may also play a role in the primer synthesis initiation step [39,56,67]. Metal C is bound by the triphosphate tail of the initiating nucleotide, probably to neutralise the negative charge and repulsive forces exerted by the tail on the neighbouring elongating nucleotide and stabilises the tail interactions with Pri2 CTD.

## Primer extension

In the second step of the biphasic synthesis mechanism, the dinucleotide is extended to form a functional primer [52]. This polymerisation step of primer synthesis occurs rapidly [34]. Polymerisation is conducted with the same functional residues as dinucleotide synthesis, whereby the 3′ primer terminus occupies the former I-site and the nucleotide to be added binds to the E-site [36]. Therefore, extension reactions also utilise the same two-metal ion mechanism of catalysis, with identical sugar selectivity as the E-site nucleotide used during dinucleotide synthesis.

Primer extension by the replicative primase can be conducted by the Pri1 subunit alone [39,52,72]. However, the rate and stability of this reaction are significantly increased by the Pri2 subunit. It has been suggested that the interaction between the CTD of Pri2 and the triphosphate group of the initiating nucleotide, which is maintained following initiation, stabilises the primase-primer:template complex [55,64]. The CTD of Pri2 is linked by a long flexible linker to the N-terminus, which in turn interacts with Pri1 [73]. The flexibility provided by the linker is required for the efficient elongation of primers. In the proposed ‘hinge’ model of primer synthesis, the linker allows the primase complex to be tethered to both the 5′- and 3′- termini of the primer during the initiation and elongation steps [55]. The archaeal primase *Ss*PriSLX is thought to operate using a similar mechanism [56,57]. PrimPol is a more efficient polymerase when its zinc finger domain is disrupted, suggesting that an interaction with the 5′ end of the primer may limit progression during primer extension [26,40,59]

## Primer termination and hand-off

Primases typically synthesise primers of a defined length, known as unit-length primers, which are suitable to hand off to a polymerase for further elongation [19]. For example, the human replicative primase Pri1 typically synthesises RNA primers of 9 nucleotides in length before the extending primer terminus is transferred to Pol α [55]. Notable exceptions do exist, such as the archaeal primases, which have evolved to synthesise longer tracts of dNTPs to fulfil a polymerase-like role in bulk genome and plasmid replication [5,7,74].

In the ‘hinge’ model proposed for human primase, the length of primers is controlled by the tethering of Pri2 CTD to the 5′-terminus of the nascent primer. Following initiation, Pri1 and the N-terminus of Pri2 extend away from Pri2 CTD, following the helical path of the nascent duplex [55]. This process continues unhindered for the first eight nucleotides of the primer. However, upon insertion of a ninth nucleotide, a steric hindrance arises between the two terminal subunits of Pri2. The resulting configuration prevents proper alignment of the terminal 3′-hydroxyl group with the phosphates of an incoming dNTP, thereby preventing further elongation. The mechanism of termination is coupled with the hand-off to Pol α, which can only occur once a primer reaches nine nucleotides in length. The catalytic subunit of Pol α interacts with Pri2 and the movement of Pri2 CTD, during primer elongation, repositions the catalytic core of Pol α into a favourable position for an intramolecular transfer of the 3′-terminus of the primer into the active site of the polymerase and subsequent primer extension [55]. This hand-off occurs rapidly, without dissociation of the complex from the DNA/RNA duplex [52,75].

The more recently proposed ‘competition’ model suggests the primase-to-polymerase switch is driven by a competition between Pri1 and Pol α for the primer 3′-end, where Pri2 CTD serves as a hub [76]. In this model, the steric collision between the Pri2 N-terminal domain and Pri2 CTD acts only as an emergency break to ensure that longer RNA primers are not produced. The structural data demonstrate that Pol α can bind to an RNA primer:Pri2 CTD complex when the primer is at least 7 nucleotides in length. Transfer of shorter primer products by Pri2 CTD to Pol α is sterically blocked.

A ‘hinge’ model of primer synthesis has been proposed for *Ss*PriSLX, whereby PriX forms the 5′ tether and defines the primer length of 14–18 nucleotides [61]. The mechanism of counting is dependent on both PriX and the presence of a 5′-triphosphate. In these organisms, which lack Pol α, an RNA primer cannot be directly extended by the replicative polymerase [77]. A solution for this arises when the presence of dNTPs in the cellular environment is accounted for. *Ss*PriSLX does not select for the sugar group of the E-site nucleotide [61]. Therefore, at physiological concentrations of nucleotides, dNTPs can be incorporated irregularly. It has been proposed that the incorporation of a single dNTP encourages dissociation of the primase complex and provides a substrate that can be extended by the replicative polymerase [77].

A unit length for eukaryotic PrimPol primers has not yet been defined. While PrimPol is proposed to remain bound to the 5′-triphosphate during primer elongation, long primer products are still observed *in vitro* [40]. In contrast with the replicative primases, PrimPol is tethered to DNA by an interaction with replication protein A (RPA), which may also provide a mechanism for limiting primer length [78]. However, long products have still been observed in primase reactions containing both PrimPol and RPA. This does not necessarily represent a single processive primer extension step, since the influence of multiple binding and extension steps was not accounted for. Therefore, further studies are required to assign a unit length for PrimPol-dependent priming *in vivo*. Additionally, the mechanism for the hand-off of PrimPol primers to replicative polymerases is currently unknown.

## Conclusions

**Figure 4:**
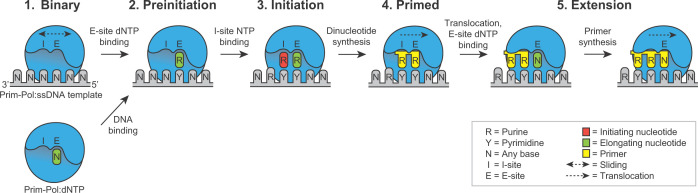
Model of the mechanism of primer synthesis Primer synthesis begins with the formation of a binary complex; either Prim-Pol:DNA or Prim-Pol:nucleotide (green; N). Next, a preinitiation complex Prim-Pol:DNA:R is formed, with a purine nucleotide (green; R) in the E-site. A purine NTP (red; R), is then bound in the I-site, creating a stable primer initiation complex to enable dinucleotide synthesis (yellow). Following turnover, the DNA binding affinity of the Prim-Pol domain reduces to allow translocation of the dinucleotide primer, whereby the 3′ terminus is translocated into the I-site/primer-binding site of the Prim-Pol domain. DNA binding affinity increases again upon the binding of an incoming nucleotide (green; N) in the E-site, enabling primer extension.

Combining the findings of extensive biochemical, biophysical, and structural studies on Prim-Pol enzymes, from a variety of organisms, reveals overt similarities in the mechanisms employed during *de novo* primer synthesis. These commonalities suggest a unified model (summarised in Figure 4) in which the fundamental aspects of primer synthesis by Prim-Pols are conserved throughout evolution and across all domains of life. These similarities begin with a preferential sequence for primer initiation. All Prim-Pols examined to date display a distinct preference for conducting dinucleotide synthesis with two purine nucleotides. Furthermore, a stimulatory effect of a purine base in the -2 template position is recurringly observed for these replicases. These preferences likely arise from a combination of strong G-C Watson–Crick base pairings and a network of stacking interactions that are best stabilised by purine nucleotides [39].

All of these primases possess I-site and E-site nucleotide binding sites within the catalytic core of their Prim-Pol domains. The well-defined E-site makes many contacts with its respective nucleotide and, accordingly, interactions between Prim-Pol enzymes and their E-site nucleotides have been detected. In contrast, interactions with the I-site nucleotide are believed to be weak and transient. Therefore, to support efficient priming, this nucleotide is likely stabilised in the active site by ancillary domains or subunits, outside of the catalytic core. This may function to control primer initiation and, therefore, regulate priming in cells. Following the generation of a unit-length primer, the mechanism of primer termination and hand-over likely differs between Prim-Pols, depending on the specialised functions that a particular enzyme has evolved to conduct in cells.

An ever-growing number of available structures has provided unprecedented insights into the *modus operandi* of Prim-Pol primases [36,37,39,55]. However, the insights provided by these structural studies are limited by the absence of either accessory domains or key substrates in the elucidated complexes. The structural determination of full-length Prim-Pols in complex with their requisite associated subunits, captured in all stages of their catalytic cycles, will undoubtedly improve our understanding of the priming process, particularly with regard to the roles that ancillary modules and domains play in primer initiation, elongation, and termination.

## Open Access

Open access for this article was enabled by the participation of University of Sussex in an all-inclusive *Read & Publish* agreement with Portland Press and the Biochemical Society under a transformative agreement with JISC.

## Abbreviations

CAPP: CRISPR-associated Prim-Pol
CTD: C-terminal domain
DSB: double-strand break
E-site: elongation site
HSV-1: Herpes simplex virus type 1
I-site: initiation site
MMEJ: microhomology-mediated end-joining
NHEJ: non-homologous end-joining
Prim-Pol: Primase-Polymerase
RPA: replication protein A
RRM: RNA recognition motif
ssDNA: single-stranded DNA
TLS: translesion synthesis
TOPRIM: topoisomerase-primase
